# Dual Task Performance in Parkinson’s Disease

**DOI:** 10.3233/BEN-110238

**Published:** 2013

**Authors:** Jennifer A. Foley, Reiner Kaschel, Sergio Della Sala

**Affiliations:** ^1^Department of NeuropsychologyNational Hospital for Neurology and NeurosurgeryQueen SquareLondonUK; ^2^Human Cognitive NeuroscienceUniversity of EdinburghEdinburghUK; ^3^Centre for Cognitive Ageing and Cognitive EpidemiologyDepartment of PsychologyUniversity of EdinburghEdinburghUK; ^4^Department of PsychologyUniversity of OsnabrückOsnabrückGermany

**Keywords:** Parkinson’s disease, Alzheimer’s disease, dual task

## Abstract

Several studies have found dual tasking to be impaired in Alzheimer's disease (AD), but unaffected by healthy ageing. It is not known if this deficit is specific to AD, or also present in other neurodegenerative disorders that can occur in later life, such as Parkinson's disease (PD). Therefore, this study investigated dual tasking in 13 people with PD, 26 AD and 42 healthy age-matched controls. The people with AD demonstrated a specific impairment in dual tasking, which worsened with increasing disease severity. The people with PD did not demonstrate any deficits in dual tasking ability, when compared to healthy controls, suggesting that the dual task impairment is specific to AD.

## 1. Introduction

### 1.1. *Dual tasking*

Dual tasking is the ability to perform two tasks at once. The cognitive processes involved in performing a dual task are thought to depend upon the nature of the two constituent tasks. When the two tasks involve similar processes (e.g. verbal processing), they are thought to compete for the same, limited, set of cognitive resources, leading to a bottleneck in processing and large dual task decrement [39,53]. However, when these two tasks involve separate processes (e.g. verbal and visuo-spatial processing), the two tasks are thought to be performed in parallel, resulting in no cognitive conflict and minimal dual task decrement [15,21–24,26,32,41]. This latter type of dual task is thought to rely upon a coordination function, which is one of the executive resources available within working memory [7].

Several studies have found dual tasking to be impaired in Alzheimer's disease (AD), but unaffected by healthy ageing. It is not known if this deficit is specific to AD or also present in other neurodegenerative disorders that can occur in later life, such as Parkinson's disease (PD). In this study, we compare dual tasking in healthy ageing, AD and PD.

### 1.2. *Dual tasking in healthy ageing*

The effect of age on the ability to dual task also appears to depend upon the nature of the two tasks. When the two tasks compete for the same cognitive resources, the observed dual task decrement increases with age [27,32,43,49]. When there is no such cognitive conflict, some studies have also reported that dual task performance declines with increasing age [3,18,19, 36]. However, in these studies, the demands of the two individual tasks were not individually calibrated to the ability of each participant. If single task performance is not equated across age groups, any group difference in dual tasking ability may simply reflect an amplification of baseline differences in the ability to perform either of the two individual tasks (c.f. [55]). Several studies have found that when the demands of the two tasks are calibrated to the ability of each individual, and single task performance is equated across groups, dual tasking ability is unaffected by normal ageing [8,9,22,23, 37,54].

ISSN 0953-4180/13/$27.50 © 2013 – IOS Press and the authors. All rights reserved

### *1.3. Dual tasking in Alzheimer's disease*

In addition to their early deficits in episodic memory, people with AD show a striking impairment in dual tasking ability [6,8,21,24,33,37,40,41,46–48,56]. As dual tasking is unaffected by healthy ageing, but is significantly impaired in AD, dual tasking impairment appears to be specific to AD. This impairment is indeed present not only in people with familial AD but also in asymptomatic carriers, when it is detectable even before the onset of the disease [42]. The deficit becomes even more pronounced with increasing disease-severity [5,6]. This finding has been replicated using various dual task combinations, including memory and motor tasks (e.g. [8]), visual and verbal memory tasks (e.g. [41]), and more everyday task combinations, such as walking and talking [14], and keeping track of who said what in a conversation [1].

Importantly, the dual task impairment in AD does not seem to reflect a general effect of increased cognitive load on a damaged brain. Logie et al. [37] systematically varied the demands of two individual tasks, and a dual task, in healthy younger and older people, and people with AD. They found that whereas all groups showed decreased performance with increasing demands, the AD group always showed a dual task impairment, even when the demands were very low. Moreover, the AD patients did not show differential sensitivity to level of demand. This suggests that the dual task impairment does not simply reflect reduced general resources, but a specific impairment in the coordination of two independent tasks.

This dual task impairment is not present in other disorders than can mimic AD, such as chronic depression or in old age [34], or Mild Cognitive Impairment (MCI) [28]. This suggests that the impairment is specific to AD, which would have both theoretical and clinical implications. However, before such specificity is assumed, it is essential to determine how dual tasking ability is affected by other neurodegenerative disorders that can occur in later life, such as Parkinson's disease (PD).

### *1.4. Parkinson's disease*

PD affects around 7 million people worldwide [52]. The disease is caused by degeneration of dopamine producing cells, initially restricted to the substantia nigra pars compacta, but later progressing to the ventral striatum and prefrontal cortex. Its characteristic motor symptoms are slowed movement or 'bradykinesia', muscular rigidity, tremor and postural instability. The presence of non-motor symptoms is also increasingly recognised, including cognitive and emotional dysfunction [30].

Studies examining the cognitive deficits in PD tend to report mainly a dysexecutive profile, but with a variable pattern of impairment across the different executive functions [45]. Several studies have reported impaired performance on measures of shifting, such as the CANTAB ID/ED [16,51,58] and other task switching paradigms [13,35]. Impairments in inhibition have also been found, with reports of poorer performance on the Hayling Sentence Completion [10,57] and Stroop tests [45]. Some studies have reported impaired performance on measures of updating, such as digit span [29], spatial span [17,60] and verbal fluency [45], but others have not [25]. There is also evidence of preserved planning abilities [45,47,48]. For a recent review, see Kehagia et al. [35].

### *1.5. Dual tasking in Parkinson's disease*

It is unclear if people with PD display deficits in dual tasking. Several studies have reported that people with PD show impaired dual tasking ability when compared to age-matched healthy controls (see Table 1 for summary of relevant studies), but these studies have used task combinations which compete for the same cognitive resources, involved baseline differences in single task performance and no calibration of the demands of the two individual tasks [12,50,59]. Accordingly, Wu and Hallett [59] found that the PD group were differentially sensitive to greater dual task complexity, which lends support to the alternative hypothesis that any apparent dual task impairment is actually an artefact of greater demand placed upon a damaged cognitive system, rather than reflecting a specific impairment in the ability to coordinate the performance of two independent tasks.

To date, there has only been one study published that has attempted to address these methodological issues. Dalrymple-Alford et al. [20] assessed eight people with PD and eight age-matched healthy control participants using a dual task paradigm based upon that initially described by Baddeley et al. [8]. Importantly, this dual task involved two tasks that loaded upon separate cognitive resources and allowed for the demands of the two tasks to be individually adjusted for each participant. The two tasks were a tracking task and a digit recall task. In the tracking task, participants were asked to use a joystick to keep an orange cross (10 mm $\times$ 10 mm)

Table 1
Studies investigating dual tasking in PD

| Study | Participants | Dual tasks | Findings | Issues with methodology |
|---|---|---|---|---|
| Brown and Marsden [12] | 17 PD, 17 age-matched controls | Stroop task plus either: random number generation (RNG), articulatory suppression or foot pressing | PD show impaired dual task performance | 1. Stroop, RNG and articulatory suppression are all verbal tasks<br>2. Baseline differences in single task performance<br>3. No calibration. |
| O'Shea, Morris and Iansek [50] | 15 PD, 15 age-matched controls | A walking task plus either: a motor task or a backwards counting task | PD show impaired dual task performance | 1. Walking and motor tasks are both visuo-motor tasks<br>2. Baseline differences in single task performance<br>3. No calibration. |
| Wu and Hallett [59] | 12 PD, 12 age-matched controls | A finger movement task (simple or complex) plus either: a tapping task or a visual letter counting task | PD show impaired dual task performance, with increasing impairment on more complex tasks | 1. Finger movement and tapping tasks are both visuo-motor tasks<br>2. Baseline differences in single task performance<br>3. No calibration |
| Dalrymple-Alford, Kalders, Jones and Watson [20] | 8 PD, 8 age-matched controls | Tracking and digit recall tasks | PD showed impaired dual task performance | 1. No overall measure of dual tasking ability<br>2. PD patients not screened for dementia |

inside a green square (starting size of 40 × 40 mm). If the participant could keep the cross within the square for 40–60% of a 20 second trial, and in three trials in total, then the size of the square decreased. This was continued until the participants could no longer keep the cross on target for 40–60% of the time, over three trials. The minimum size at which the participant could remain on target for at 40–60% of the time was considered to be their tracking span. In the digit recall task, participants were asked to listen to and repeat back sequences of digits. If the participant could recall the digits correctly, in two out of three trials, the length of the sequence was increased by one digit. This was continued until the participant could no longer recall two out of three digit sequences correctly. The maximum sequence length that the participant could recall accurately was considered to be their digit span. They then adjusted the demands of the tracking and digit recall tasks according to the participant's span, and compared performance when the two tasks were performed alone and then together.

The PD patients demonstrated significantly lower proportional performance of the tracking task than the healthy controls, but no difference in proportional performance of the digit recall task. Thus, it appears that the PD group show difficulties performing dual tasks. However, closer examination of the data suggest that the PD patients actually performed better (yet not statistically) than the healthy controls on the digit recall task in both single and dual task conditions. Therefore, it is possible that if dual task performance on these two measures was combined, any trade-off between tasks would be accounted for and a group difference might disappear. Moreover, unlike the other studies described, the PD patients were not screened for cognitive impairment, and thus it is also possible that some of the PD patients had Parkinson's disease dementia (PD-D). Indeed, like all of the healthy controls, three out of the eight PD patients demonstrated less than 10% drop in tracking performance in the dual task condition (with one actually showing a gain of 5%), whereas the other five patients showed drops of between 25 and 40%, which may suggest that only some of the patients had difficulty performing the dual task.

Therefore, it remains unclear if people with PD without dementia display dual task impairment. The aim of the current study was to compare dual task performance in people with PD, AD and healthy age-matched controls, to determine if dual tasking is also impaired in PD. In order to account for the methodological issues previously raised, this study used a dual task paradigm based upon that described by Baddeley et al. [8], but, in addition, also provided an overall measure of dual task performance to account for any possible trade-off in between performance on the two tasks. Moreover, all PD participants were screened for dementia to ensure that appropriate conclusions could be drawn.

## 2. Methods

### 2.1. Participants

A total of 13 people with PD, 26 people with AD and 42 age-matched controls participated in this study.

The 13 PD patients (six women and seven men) had been diagnosed with idiopathic PD by a consultant neurologist and assessed as being between Hoehn and Yahr stages I–III (stage I, $n = 1$; stage II $n = 10$; stage III $n = 2$). They had a mean disease duration of 69.15 months (SD = 53.09, range = 9–192). All patients were screened for dementia using the Mini-Mental Status Examination (MMSE) and scored 26 and above, with a mean score of 28.85 (*SD* = 1.28, range = 26–30). All patients were tested while on anti-parkinsonian medication. Dosages of dopaminergic medication were converted into equivalent levadopa dosages using the standard formula, as described in Brodsky, Godbold, Roth and Olanow [11], and ranged from 12–1412 mg per day (mean = 510.00 mg, *SD* = 345.54). The PD participants were aged between 55 and 74 years (mean = 66.08 years, *SD* = 6.05), and had a mean of 15.63 years of formal education (*SD* = 3.41, range = 10–23). They had no history of other neurological impairment, psychiatric disorder, major medical illness or brain injury.

The 26 AD patients (12 women and 14 men) had been diagnosed in accordance with National Institute of Neurologic, Communicative Disorders, and Stroke-AD and Related Disorders Association (NINCDS-ADRDA; McKhann et al. [44]) and DSM-IV [2] criteria. The AD patients had a mean MMSE score of 21.54 (*SD* = 3.19, range = 13–25). They ranged in age between 46 and 79 years (mean = 64.65 years, *SD* = 8.61) and had a mean of 10.12 years of formal education (*SD* = 2.64, range = 8–18). They had no history of other neurological impairment, psychiatric disorder, major medical illness or brain injury.

The 42 healthy controls (26 women and 16 men) were matched as closely as possible to the PD and AD groups for age, gender and education. They had a mean MMSE score of 28.76 (*SD* = 1.27, range = 26–30). The healthy participants ranged in age between 46 and 82 years (mean = 63.21, *SD* = 8.24) and a mean of 10.88 years of formal education (*SD* = 3.26, range = 8–21). All healthy participants had no history of psychiatric, neurological or degenerative disorder, or brain injury.

The research was done in accordance with the Helsinki declaration, and had ethical approval from NHS Lothian and University of Edinburgh research ethics committees.

### 2.2. Procedure and materials

Each participant completed the dual task assessment, which consisted of performing digit recall and tracking tasks separately and then simultaneously. This dual task assessment, and the full instructions for its administration and scoring, can be found at www.psy.ed.ac.uk/people/sdsala/tests/sdsdualtask/.

Before commencing the digit recall task, digit span for each individual was established. Participants heard a list of digits at a rate of one per second. Participants were then asked to repeat these digits back in the same order as they heard them. The initial sequence length was two digits long and participants were presented with six sequences at each sequence length. If five out of the six sequences were recalled correctly, the digit sequence was lengthened by one digit. Once a participant could no longer recall five out of the six digit sequences, digit span was taken as the maximum length at which the participant was able to recall five out of six digit sequences correctly. After the participant's span had been established, they heard sequences at their individual span length for immediate serial recall, and this was repeated for as many sequences as could be presented and recalled over a 90 second period. Therefore, the number of lists for each participant varied depending on the length of their digit span, and the performance measure was the proportion of digits accurately recalled in the correct serial order position.

The tracking task consisted of using a pencil to draw a line through a series of circles arranged in a path around a sheet of A3 paper. Participants were given a shortened version for a practice trial, with only 17 circles, to ensure that they understood the task demands. After this, the participant was presented with the full version comprising 319 circles, and asked to start at one end of the path and draw a line through each successive circle as quickly as they could for a period of 90 seconds. The performance measure was the number of circles crossed by the pencil.

In the dual task condition, participants were asked to perform the tracking task at the same time as listening to and repeating back the digit sequences they heard, for a further 90 seconds. The performance measures were the proportion of digits accurately recalled in the correct serial order position and the number of circles crossed by the pencil. Proportional performance in digit recall ($p$m) was calculated by measuring the change in digit recall between single ($m$single) and dual task ($m$dual) conditions, where $m$ is the proportion of digits recalled accurately, and using:

$$p_{\mathrm{m}} = 100 - \frac{(m_{\mathrm{single}} - m_{\mathrm{dual}}) \times 100}{m_{\mathrm{single}}}$$

Proportional performance in tracking ($p$t) was calculated by measuring the change in tracking between single ($t$single) and dual task ($t$dual) conditions, where $t$ is the number of circles drawn through, and using:

$$p_{\mathrm{t}} = 100 - \frac{(t_{\mathrm{single}} - t_{\mathrm{dual}}) \times 100}{t_{\mathrm{single}}}$$

In order to account for any possible trade-off in performance across tasks, performance on both tasks was combined to give a measure of overall dual task performance ($\mu$), which was calculated by using:

$$\mu = \frac{p_{\mathrm{m}} + p_{\mathrm{t}}}{2}$$

## 3. Results

### 3.1. Analytical strategy

Mean and standard deviations were calculated for each of the variables. Normality of distribution was assessed using the Kolmogorov-Smirnov test and, if significant, by examining the $z$-scores for skewness and kurtosis. Homogeneity of variance was assessed using the Levene's test. Unless otherwise stated, all data met the assumptions of normality and homogeneity of variance. Data were analysed using PASW Statistics Data Editor.

### 3.2. Participants

A one-way between-subjects analysis of variance (ANOVA) showed that there were no group differences in age [$F$(2, 78) = 0.70, n.s]. A Kolmogorov-Smirnov test revealed that education was not normally distributed in two of the three participant groups. There was a positively skewed and leptokurtic distribution of years of education in the AD [$D$ (26) = 0.36, $p$ < 0.001; $_z$skewness = 4.65; $_z$kurtosis = 4.22] and healthy groups [$D$ (42) = 0.37, $p$ < 0.001; $_z$skewness = 5.14; $_z$kurtosis = 3.74]. A Kruskal-Wallis test revealed significant group differences in years of education [$H$ (2) = 20.30, $p$ < 0.001], and Mann-Whitney tests revealed that the PD patients were significantly more educated than both the AD patients ($U$ = 29.00, $p$ < 0.001) and the healthy participants ($U$ = 71.50, $p$ < 0.001), but there was no difference between the AD and healthy groups ($U$ = 419.50, n.s.).

Table 2
Group means and standard deviations of proportional performance in digit recall ($p$m), tracking ($p$t) and both tasks overall ($\mu$)

| | PD<br>Mean (SD) | AD<br>Mean (SD) | Healthy<br>Mean (SD) |
|---|---|---|---|
| Digit recall | 83.21 (18.07) | 92.51 (10.95) | 93.71 (10.01) |
| Tracking | 95.53 (19.95) | 64.52 (24.04) | 90.12 (12.50) |
| Overall | 89.37 (15.33) | 78.50 (2.73) | 91.92 (7.36) |

A Levene's test revealed that the three participant groups had MMSE scores with significant different variances [$F$ (2, 78) = 21.16, $p$ < 0.001]. A Kruskal-Wallis test revealed that there was a significant difference in mean MMSE score between the three groups [$H$ (2) = 54.85, $p$ < 0.001]. Mann-Whitney tests showed that the AD participants had lower MMSE scores than both PD ($U$ = 0.00, $p$ < 0.001) and healthy groups ($U$ = 0.00, $p$ < 0.001), but with no difference between the PD and healthy groups ($U$ = 263.50, n.s.). Years of education was not found to be related to MMSE score [$F$ (1, 76) = 2.41, n.s.].

### 3.3. Digit span

A one-way ANOVA revealed a significant group difference in digit span [$F$ (2, 76) = 28.45, $p$ < 0.001]. Years of education was found to be related to digit span performance [$F$ (1, 76) = 19.71, $p$ < 0.001], but when the effect of education was partialled out, using an analysis of co-variance, there remained a group difference [$F$ (3, 76) = 28.88, $p$ < 0.001]. Bonferroni-corrected post-hoc analyses revealed that the AD group had significantly shorter digit span (mean = 4.23, *SD* = 0.95) than both the PD (mean = 7.08, *SD* = 1.50, $p$ < 0.001) and healthy groups (mean = 5.40, *SD* = 0.17, $p$ < 0.001), whose difference fell short of significance ($p$ = 0.06).

### 3.4. Single task performance

The healthy group's digit recall performance in the single task condition was significantly positively skewed [$D$ (42) = 0.20, $p$ < 0.05; $_z$skewness = 2.44]. A Kruskal-Wallis test revealed no group differences in mean digit recall performance in the single task condition [$H$ (2) = 2.35, n.s.], with no obvious outliers explaining the apparent group difference in performance.

A one-way ANOVA revealed a significant group difference in tracking performance in the single task condition [$F$ (2, 78) = 16.62, $p$ < 0.001]. Bonferroni-corrected post-hoc analyses revealed that healthy adults performed better on the tracking task than both the PD

and AD groups (both $p < 0.001$), but there was no difference between the two patient groups (n.s.).

Years of education was not found to be related to either digit recall [$F$ (1, 76) = 0.78, $p$ = 0.38] or tracking performance [$F$ (1, 76) = 0.60, n.s.].

### 3.5. *Dual task performance*

Group means and *SD*s of proportional performance in digit recall ($p$m), tracking ($p$t) and both tasks overall ($\mu$) are presented in Table 2.

The score distribution of the healthy group's proportional performance of the digit recall task ($p$m) was significantly negatively skewed and leptokurtic [$D$ (42) = 0.17, $p < 0.05$; $_z$skewness = −3.68; $_z$kurtosis = 3.11]. A Levene's test also revealed that the three participant groups had significantly different variances on this task [$F$ (2, 78) = 9.14, $p < 0.001$]. A Kruskal-Wallis test revealed no group differences in mean proportional performance of digit recall [$H$ (2) = 3.34, n.s.].

A Levene's test also revealed that there were significant differences in the variances in scores of proportional performance of tracking ($p$t) between the three groups [$F$ (2, 78) = 26.89, $p < 0.001$]. A Kruskal-Wallis test revealed a significant group difference in these scores [$H$ (2) = 25.89, $p < 0.001$]. Mann-Whitney tests showed that the AD group had poorer proportional performance of tracking than both the PD ($U$ = 47.50, $p < 0.001$) and healthy groups ($U$ = 165.00, $p < 0.001$), but no difference between the PD and healthy groups ($U$ = 259.50, n.s.).

Wilcoxon signed-rank tests revealed that the difference in the overall dual task decrement shown between the digit recall and tracking tasks in either the PD ($z = -1.73$, $p = 0.08$) or healthy groups ($z = -1.73$, $p = 0.10$) did not reach significance. However, the AD group showed significantly greater dual task decrement in the tracking task than the digit recall task ($z = -4.31$, $p < 0.001$).

A Levene's test also revealed that there were significant differences in the variances of overall dual task performance ($\mu$) between the three groups [$F$ (2, 78) = 10.08, $p < 0.001$]. A Kruskal-Wallis test revealed a significant group difference in these scores [$H$ (2) = 18.89, $p < 0.001$]. Mann-Whitney tests showed that the AD group had poorer overall performance than both the PD ($U$ = 100.00, $p < 0.005$) and healthy groups ($U$ = 203.00, $p < 0.001$), but with no difference between the PD and healthy groups ($U$ = 210.00, n.s.)[1].

Spearman's correlations revealed proportional performance of both tasks overall was not associated with years of education ($\rho = 0.14$, n.s.), but was significantly correlated with MMSE score ($\rho = 0.47$, $p < 0.001$).

A Pearson's correlation revealed that PD patients' overall dual task performance was not associated with the duration of their PD ($r = -0.21$, n.s.). The PD patients' equivalent levadopa dosages had a leptokurtic distribution [$D$ (13) = 0.30, $p < 0.05$; $_z$kurtosis = 2.92] and a Spearman's correlation revealed that dosage was not associated with overall dual task performance ($\rho = 0.33$, n.s.).

## 4. Discussion

Several studies have reported that dual tasking is unaffected by healthy ageing but significantly impaired in AD [6,8,21,24,28,33,37,40–42,56]. In this study, the people with AD were significantly impaired in dual tasking when compared with healthy age-matched controls, confirming this previously reported pattern of impairment.

### 4.1. *Alzheimer's disease*

The main aim of the present study was to determine if dual tasking impairment was specific to AD, or also affected by another neurodegenerative condition, namely PD. When people with PD were compared with people with AD and healthy age-matched controls, only the people with AD demonstrated dual task impairment. Dual tasking ability was negatively correlated with increasing disease severity. These findings are in line with those of previous studies reporting that dual tasking is specifically impaired by AD, and not sensitive to other disorders that can mimic AD, such as chronic depression in old age [34], or MCI [28].

Furthermore, the AD group demonstrated significantly shorter digit spans than the other two groups, with the PD group appearing to have longer digit spans than the healthy controls. It is possible that the differing digit spans in three groups simply reflects the differing number of years of formal education received.

More importantly, perhaps, is that this variation in digit span performance underlines the need to adjust the demands of the tasks according to such individual differences. Indeed, when the level of demand was individually titrated, there were no group differences in single task digit recall performance, confirming that the dual task impairment observed was not caused by relatively poorer performance on the single tasks, but rather reflects a specific impairment in the ability to coordinate the simultaneous performance of two independent tasks.

The finding that dual tasking is preserved in PD contradicts previous research [12,20,50,59]. However, these previous studies have used two tasks that do not load upon separate cognitive resources, do not equate single task performance across groups and/or do not take into account possible trade-off in dual task performance. This study is the first to show, that when care is taken to avoid these methodological confounds, that people with PD do not show dual task impairment. The sparing of dual task ability is consistent with the previous literature reporting a patchy pattern of impairment across different cognitive functions in PD (e.g. [45]), with deficits found in set-shifting (e.g. [16]) and inhibition (e.g. [57]), but less clear impairments observed in updating (e.g. [25]) and planning abilities (e.g. [45]).

The lack of dual task impairment in PD cannot be explained by the relative differences in education (or digit span), as although the PD patients were, on average, more educated than the other two groups, education was not related to dual task performance. Dual task performance in the PD group was also not associated with either disease duration or equivalent levadopa dosage. Previous studies have also found few correlations between cognitive performance and disease duration and/or motor symptoms (e.g. [45]). It is possible that the sparing of dual tasking ability in PD is limited to people without PD-D. The previous study by Dalrymple-Alford et al. [20] that found dual task deficits in people with PD did not screen for possible dementia. The cognitive phenotype of PD-D is still being determined [30], and dual tasking ability in this type of dementia has yet to be examined.

This specificity is of considerable theoretical and clinical interest. It appears that the ability to coordinate performance of two independent tasks is a function available in the healthy brain, but specifically damaged by AD, leading to further questions about its underlying biological mechanisms, and the neurobiological underpinnings of dual tasking.

The lack of dual task impairment in depression [34], MCI [28], and now also PD, suggests that dual task assessments may be particularly useful in the differential diagnosis of AD. Indeed, the paper-and-pencil version used in this study offers an easily-transportable, inexpensive and usable measure, which could be readily used in primary health care to support the early and accurate diagnosis of AD. Dual task measure are also appropriate for following up people over time, as dual task performance is associated with disease severity, but less vulnerable to floor effects than measures of episodic memory (e.g. [31]).

## Acknowledgments

This work was funded by an award from the University of Edinburgh Moray Endowment Fund awarded to Jennifer A. Foley. We are very grateful to Parkinson's UK for their assistance with recruitment and John Howard and Rebecca Sanderson for their help with testing the PD patients.
